# Design of a Digital 3D Model of Transport–Technological Environment of Open-Pit Mines Based on the Common Use of Telemetric and Geospatial Information

**DOI:** 10.3390/s21186277

**Published:** 2021-09-18

**Authors:** Igor Temkin, Alexander Myaskov, Sergey Deryabin, Iliya Konov, Alexander Ivannikov

**Affiliations:** National University of Science and Technology MISiS, Moscow 119049, Russia; myaskov@misis.ru (A.M.); deryabin.sa@misis.ru (S.D.); haina@misis.ru (I.K.); ivannikov.al@misis.ru (A.I.)

**Keywords:** digital twin, autonomous haulage systems, open-pit mine, multiagent systems, tessellation paving, geospatial information, telemetry, trucks, survey

## Abstract

This article is devoted to the issues of processing and analysis of heterogeneous information related to the functioning of mining transport equipment, which becomes available for analysis within the framework of modern technological operations control systems in open-pit mines. These issues are very relevant to robotized technological operations. The paper gives a brief overview of the modern landscape of the autonomous haulage systems management problems, the features of the platform approach to solving the problem of managing unmanned transport and technological processes in open pits are considered. The concept of an agent-based approach to the modeling of an open-pit mining is described in detail on the basis of the interaction of three systems: technical, infrastructural–technological, and geostructural. Some features of the developed platform architecture integration of heterogeneous information are discussed. The principles of information integration are considered in detail when constructing a dynamic 3D model (digital twin) of infrastructure and technological system elements using large arrays of telemetric data. The results of building digital models of open-pit technological roads are presented. The resulting models are comparatively analyzed in the process of optimizing of the interaction of technical autonomous mobile agents and elements of technological infrastructure.

## 1. Introduction

The main trend in the development of open-pit mining technologies today is the use of robotic and autonomous intelligent mining machines and devices. The efficiency and degree of environmental safety of mining operations in open pits mainly depend on the organization of the functioning of the mining and transport equipment, which includes excavators for various purposes, heavy-duty dump trucks, as well as other specialized vehicles. Since the mid-1960s, methods and models of planning and optimization of open-pit mining, as well as dispatch control of mining and transport processes, have been actively developing. In recent years, a number of projects have been developed successfully. In recent years, number of autonomous rock mass haulage systems in the open-pit mines have been implemented around the world [1,2,3,4]. Among the most famous projects are the Australian quarry of Rio Tinto in the Pilbara region, where in 2015, with the participation of Komatsu, a fully autonomous robotic transport equipment consisting of 150 dump trucks was applied for the first time. Further in that context, the well-known projects of some Russian companies may be noted [5,6,7].

**Figure 1:**
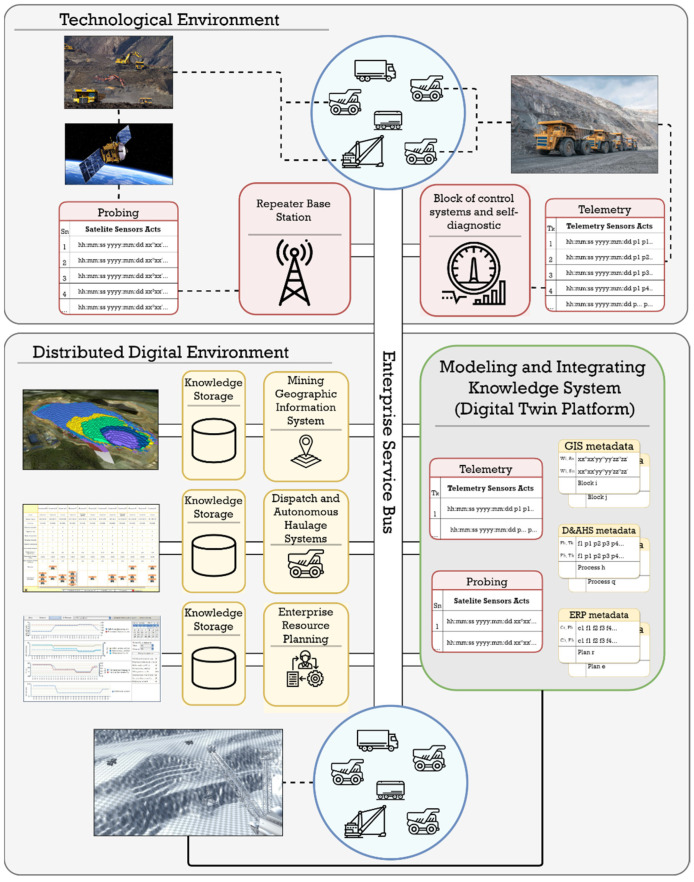
Simplified diagram of the organization of transport and technological processes using robotic and autonomous mining equipment.

There are fundamental changes in the information environment when making control decisions connected with the introduction within the framework of constantly developing automated control system production processes in mines of various technical sensor devices, as well as various telecommunication systems [4,5,8,9,10]. The continuous movement of robotic dump trucks across all production areas of the mining enterprise allows collecting information not only about the object itself (dump truck), but also about the surrounding technological environment. The inclusion of robotic objects equipped with a set of sensors of various types in the mining transport system leads to a sharp increase in the volume of spatiotemporal data entering the system through telemetry. Figure 1 shows a diagram that provides a general idea of the landscape, against the background of which the tasks discussed in this article are solved.

**Figure 2:**
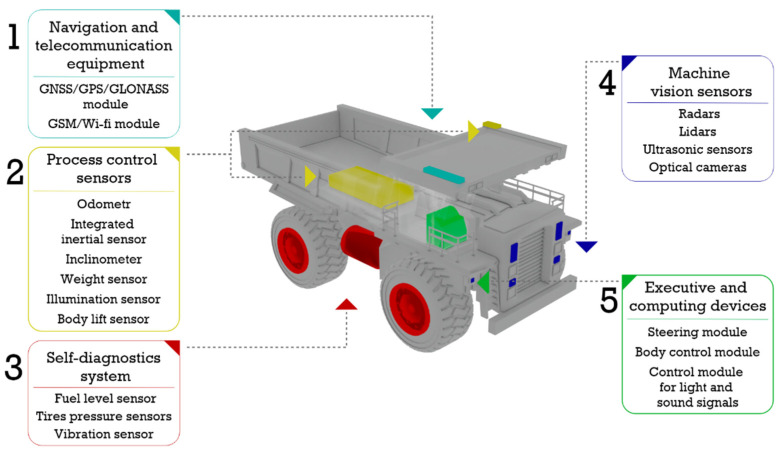
Set of sensor equipment of a robotic heavy-duty dump truck.

At the current moment, most of the mining enterprises already have automated control systems for transport and mining equipment, which ensure the support of the dispatching during extraction of mineral raw materials. The main functions of these systems include collecting telemetry from equipment (satellite coordinates, speed, cargo weight, fuel level, etc.); describing the technological and technical condition of the equipment; automated monitoring of indicators; and, in some cases, making management decisions (issuing shift tasks, redistribution along routes, determination of the need for maintenance or repair) [6,11]. The typical hardware composition of the onboard systems of robotic mining vehicles (schematically presented in Figure 2) includes the following:

Block 1. Navigation and telecommunication equipment (GNSS/GPS/GLONASS, GSM/Wi-Fi radio module).

Block 2. Process control sensors (odometer, integrated inertial sensor, inclinometer, weight sensor, light sensor, body lift sensor).

Block 3. Self-diagnosis sensors (fuel level sensor, tire pressure sensors, vibration sensors).

Block 4. Machine vision sensors (radar, lidars, ultrasonic sensors, optical cameras).

Block 5. Executive and computing devices (steering module, body control module, light and sound signal control module).

The requirement to ensure the promptness and accuracy of processing this data becomes extremely important, especially in the context of the development of autonomous transport systems in which unmanned transport–technological operations are carried out [12,13,14]. This is due to the fact that the movement of mobile objects in the process of transporting rock mass must be continuously adjusted paying attention to both the actual relative position of autonomous agents on quarry routes at a certain point in time and taking into account changes in the technological environment in which these mobile objects exist.

In recent years, a lot of publications concerning the functioning of autonomous transport, both in urban conditions and in an industrial environment, have appeared. Much attention in that area is paid to the use of computational models for processing and analyzing large volumes of heterogeneous information in the problems of identifying the state and predicting the behavior of transport systems in the social and industrial spheres [15,16,17]. Thus, for example, in multiagent transport systems to estimate the predicted speed of mobile objects taking into account traffic, multiple regression models and different types of autoregressive equations [18,19,20] are successfully used. In a number of other works, dealing with industrial transport systems, multiple regression models are used to form criteria for calculating optimal routes for each of the agents, taking into account not only speed modes, but also the actual state of the technological roads. Of great interest is the issue of coordinating joint actions of industrial robots moving along the earth’s surface (as a rule, these are various transport devices). A number of works are devoted to these questions, for example, references [21,22]. Most of these works investigate the interaction of the same type of robotic transport devices. However, there are practically no results of studies describing the functioning of autonomous heterogeneous industrial robotic systems in the complex dynamic environment.

Moreover, much attention is paid to the idea of using a complex of methods and models, united by the concept of “soft computing” in the problem of coordinating the movement of autonomous transport agents [2,3,23]. The advantage of this approach is the natural possibility of integrating cognitive and computational models [24] (including multilayer deep learning networks and algorithms of choice for sustainable development of mining production [25]).

In recent years, Digital Twin technologies have attracted considerable interest among researchers [7,11,13,14,26]. Although the conceptual idea of a Digital Twin (Digital Shadow)—that is, a model that completely copies the behavior of an object and, in a certain sense, even predetermines its future state—has been known for more than 15 years [27,28], opportunities of using such a system in control tasks (such as strategically and operatively for dispatching) open up today [29,30]. Further, if for discrete conveyor-line production at the moment there are mainly technical issues of implementing control using 3D models built using digital twin technologies; then, for productions with continuous and nonstationary technological cycles and with a huge fleet of different types of sensor devices, (in particular, transport and technological operations of open-pit mines apply to that type of processes), there are problems associated with the integration, and temporal and spatial scaling of heterogeneous information flows for the creation of digital twins [31,32,33,34,35,36,37].

This article discusses some of the issues related to the development of digital twins of complex spatially distributed objects (for example, open-pit mines), which are an important element of transport–technological process control platforms, based on heterogeneous sensory information. By the term platform, in the case of a quarry, we mean a complex of object-independent (invariant with respect to the geostructure of the field, development schemes, topology, and elements of the mining and transport complex of the open pit) software and hardware services and regulations that ensure their interaction.

## 2. Conceptual Architecture and Mechanisms of Functioning of an Intelligent Geoinformation Control Platform

The architecture of the platform was designed, taking into account the results of structuring the information environment (agents, functional blocks, information models) on the basis of various modeling methods: functional–structural, datalogical, physical, imitational. In the course of the research, experiments were carried out on the basis of fragments of a real object using a laboratory model of a quarry and physical models of dump trucks, as well as a previously developed prototype version of a dispatch control system, built around a computing server for processing telemetric information, generating control commands, and visualizing the operation of mobile objects.

It is known that the foundation of the technological process of open-pit mining is a complex of transport and technological operations, which include the following:

- Mineral resource excavation and loading operations at the excavator site;
- Movement along certain routes from the excavator site to the points of unloading;
- Unloading of rock mass in the special areas;
- Movement of empty dump trucks to excavator sites;
- Maneuvering on excavator sites.

It should be noted that the functioning of autonomous transport technical devices should be organized in such a way that their interaction at all stages of the technological cycle satisfy the requirements of maximum safety and efficiency in accordance with the given integral criteria. It should be considered that the technological operations implemented by autonomous devices differ significantly from stage to stage and their implementation depends on information flows generated by fundamentally different information entities. Therefore, for a sufficiently complete and, if possible, formal description of the interaction procedures—“excavator—bottomhole space”, “excavator—dump truck”, “dump truck—route”, “dump truck—dump truck”, “dump truck—road track”, “dump truck—technological zone (loading, unloading)”—we proposed dividing the entire platform functions between a number of agent systems that are obvious and natural for mining enterprises. The peculiarity of our approach is to consider each of these systems, described below, as multiagent:

$a_{1}$. Agent technical system—$A_{T}$, which includes technical agents such as mobile transport facilities (trucks, auxiliary vehicles—road vehicles for various purposes), as well as conditionally stationary mining machines (excavators, movable drilling rigs). The functioning of these agents in an unmanned version is determined by the following:

- Data of the onboard monitoring system, which provides information about the current location and technical condition of the agent;
- Tasks for execution received from the dispatching system;
- Digital models of the chosen routes;
- Sensory information obtained when interacting with other agents.

Note that the degree of autonomy, which defines a specific functioning algorithm, directly depends on the mechanisms of interaction with other agents, in particular, infrastructure agents, as well as on the functions that are delegated to these infrastructural–technological agents.

$a_{2}$. Agent infrastructure—technological system—$A_{I}$. Within the framework of this system, the technological environment is described, in particular, open-pit technological roads, quarry ledge sides, and excavator and unloading sites. Let us take a brief look at a few examples.

Excavator platform agent. Restrictions imposed by safety rules determine the scenarios of the behavior of the mobile agent and the “excavator site” agent from the moment the dump truck is submitted for loading, until it leaves the territory of the point. The entire cycle of a dump truck on the territory of the loading point consists of several phases—waiting for permission to enter the territory, waiting for permission to submit for loading, waiting for permission to leave the territory—and is described as the communication of internal scripts of two agents using the stack of corresponding protocols.

“Unloading site” agent. Similar to the previous example, the restrictions imposed by security rules determine the behavior scenarios of the mobile agent and the infrastructure agent “unloading site” from the moment the dump truck enters the territory of the point and until it leaves the territory.

Agent “road”. The dump truck is moved between loading/unloading points along technological roads. The agent “road” is characterized by a number of technological indicators. The main one among them is the digital model, which, at the level of atomic elements (primitives), forms an environment for constructing optimal trajectories for the movement of autonomous mobile objects.

In addition, some agents are provided with such attributes as the following examples:

- Current traffic intensity;
- Number of dump tracks passing through the section of the road per unit of time;
- Speed of movement for which the road is designed;
- Permissible weight and dimensions of the fleet;
- Bandwidth;
- Stability of the quarry ledge sides.

$a_{3}$. Geostructural agents are block elements that describe the structure of the geological field in the same way as in classical geographic information systems. The geometric parameters of the blocks depend on the technological characteristics of mining equipment (including, of course, size) and the required accuracy of positioning of mining machines and their individual elements (for example, an excavator bucket) determined at the stage of developing control algorithms.

As a result, on the base of the formed concept, we developed a working version of the architecture (optimization continues), which includes the following main components:

- Enterprise Service Bus is a software unified platform interface (Application Programming Interface) that supports standard data transfer protocols used in mining enterprises and Industrial Internet of Things protocols for organizing the interaction of enterprise systems, functional agents, and platform modules.
- Brokers of messages of the technological environment, which are necessary for organizing the transfer of data between agents of the same or different classes within the technological environment and their switching with the service bus of the enterprise. These smart devices should be located on stationary repeaters in the quarry, and their main purpose is to aggregate telemetry information from agents of uniform technological or geographic groups, transmit control commands from the enterprise service bus to agents, and transfer telemetry data and command execution results back to the service bus. The second task of these brokers is to organize information interaction between agents within the groups in the implementation of complex automatic operations, which involves maneuvering a dump truck when approaching an excavator loading or maneuvering when passing while on a transport site. For example, MQTT brokers provide fast and reliable transmission of measured telemetry to minimize errors in the determination of mutual approach distances, which was confirmed using laboratory physical modeling.

Intelligent brokers provide dispatching of enterprise data streams; decodes messages in different protocols and formats received by the service bus; identifies data; determines their type, target purposes (agent-based objectification), and lifetime; and forms queues and distributes data over them to transfer them to agents or systems via bus interfaces; thus, providing the next functions:

1. Automatic registration of requests and proposals for specific types of information by listening for and detecting incoming messages (data, requests, and commands) from information entities to the service bus.
2. Automatically manage information flows by sorting, grouping, and cataloging data by queue.
3. Monitoring the quality of information and feedback in order to maintain the relevance and reliability of control processes during data transmission.
4. Optimization of databases by removing redundant, inaccurate, or outdated information.
5. End-to-end modeling and visualization to ensure high-precision virtual display of the technological environment using data aggregation and integration at different time horizons (archive, real, and forecast).

To build a digital dynamic 3D model of a quarry and transport–technological processes, a set of software modules was developed, which, in our opinion, are absolutely necessary for the implementation of intelligent control of industrial haulage systems:

- Module for the automatic construction of infrastructure agents using inhomogeneous information: telemetry data, data from a mining and geological system, remote sensing systems, satellite and aerial photographs: quarry—road schemes—routes—technological zones—block model GIS.
- Module for automatic construction of mobile agents using archived and up-to-date telemetry data, technical passports, and regulatory documents: dump trucks, excavators, loaders, etc.
- Module for monitoring and predicting the operational (technical) state of functional agents, which implements an algorithm for monitoring and predicting the state of infrastructure agents (quality of the roadway, changes in the actual coordinates of edges, and terminal vertices of the route graph).
- Module for monitoring and forecasting production indicators.
- Module of intelligent control that generates adjustments of control commands for agents of the technological environment, controls and tests instances of metadata and telemetry data for transmission to enterprise systems; the monitoring and predictive analysis module in order to increase the generalizing capabilities of control models, including intelligent routing of mobile agents with multiparameter criteria optimizations and constraints.

**Figure 3:**
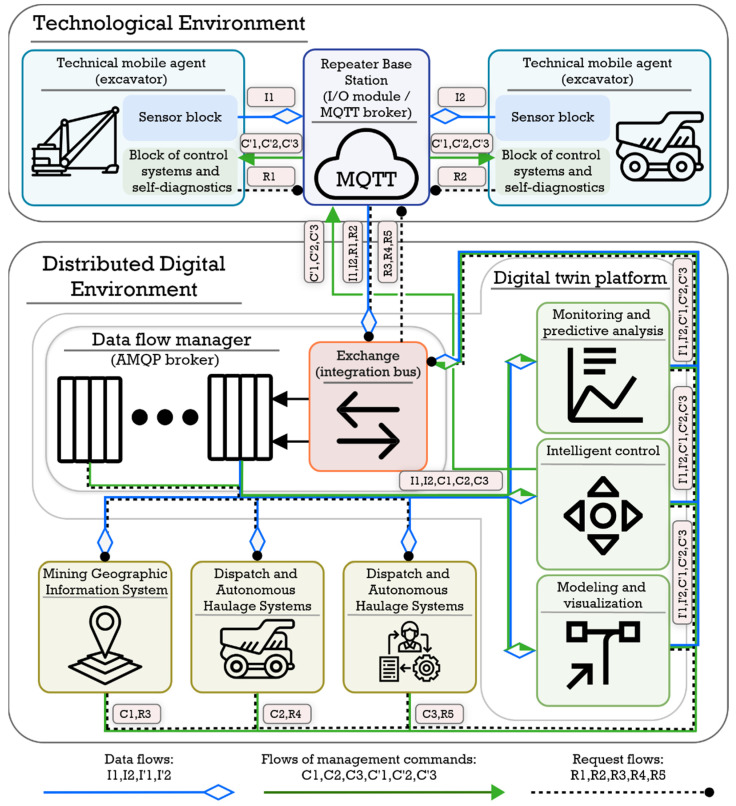
Simplified platform architecture.

Figure 3 shows the general architecture of a geoinformation platform for providing autonomous transport and technological operations of a quarry.

## 3. The Conception of the Quarry Technological Environment Dynamic Digital Model Building

A detailed description of the implemented functional and procedural tools in the previous section is necessary to demonstrate the real capabilities of the platform in data integration.

From our point of view, the certain novelty of the platform being created is the inclusion of additional functionality in order to ensure a real transition to the autonomous dispatch of technological processes that are carried out by robots. In particular, this functionality includes the following:

- Dynamic integration and aggregation of heterogeneous mining information;
- Construction of a dynamic, three-dimensional visualization of the production environment;
- Optimization modeling of technological processes.

The fundamental difference between the described functions of the platform and existing solutions already used in mining industry is the organization of continuous automatic processes for updating data and building models, as well as in the way of interpreting the results in the form of a common interactive digital technological environment that facilitates the perception of users and opens up opportunities to the construction of more-perfect control actions.

However, the integration of a mining–geological and geoinformation data into adjacent control systems is not a trivial task. On the one hand, data presentation models describing the geometric, spatial, and geological properties of the production environment are the necessary basis for subsequent decisions in the field of mining management; on the other hand, they have data of different origin that are difficult to integrate into a single digital functional structure. The difference in the nature of the origin lies both in the frequency of updating and the timing of the relevance of the data, as well as in the reliability of their sources and methods of primary acquisition. This section presents the developed procedure for constructing a digital interactive model of the main element of the infrastructure system: the transport–technological environment, including technological roads, excavator sites, and loading and unloading zones.

It is clear that control with the participation of a dispatcher robot should take into account not only the precise positioning of all objects of infrastructure and technical systems, but also the qualitative characteristics of individual elements, which are agents of the infrastructure system. Thus, an integral representation of the common infrastructure agent “quarry” is a set of points with coordinates x, y, z, located inside the “cube” with additional geoinformation features assigned to these points.

Such a representation of a digital model of a quarry is clearly redundant, and the search for optimal control parameters in such a combinatorial space is a rather complicated and time-consuming process. In this regard, it is proposed to split the original set of coordinates into some family of subsets—structurally and functionally unified atomic elements that have a full set of mining-geological and geoinformation characteristics and interact with each other.

The separation of the geoinformation space of open-pit mining into atomic elements includes two stages:

1. Dividing the infrastructure agent “quarry” into atomic elements along the surface in order to identify the main technological zones, their geometric orientation in space, and the possibility of assigning quality properties of the soil (roadbed).
2. Dividing the open pit into the depths in the form of a block model to determine the geological composition of the field and the accompanying geophysical properties of individual infrastructural parts.

Our main attention is focused on building digital models of quarry technological zones—first of all, technological roads. The importance of this task is explained by the fact that the safe and rational movement of mobile objects in an unmanned version is ensured by a precise interaction of agents belonging to the $A_{T}$ and $A_{I}$ systems.

When developing an algorithm for dividing the surface part of the open-pit space into atomic elements (primitives), the sources of information were preliminarily determined for forming and updating the geospatial structures of the open pit with the greatest accuracy and completeness. As such, we consider the following sources:

- Results of mine-surveying;
- Satellite or aerial photographs of the area;
- Telemetry measurements of onboard mining equipment.

**Figure 4:**
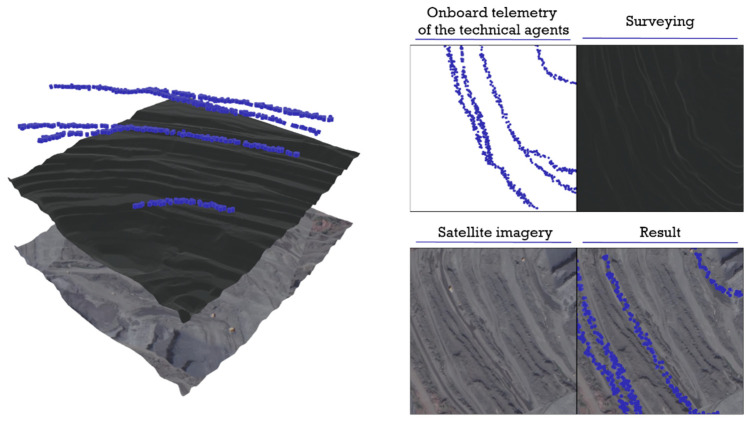
Three-layer representation of geospatial data and the type of results obtained.

Each of the listed sources forms an isolated data layer describing the geospatial position of the technological zones of the quarry and provides information similar in its properties, but different in the physical nature of the measurement. The general layer-by-layer representation of geospatial data, obtained as a result of processing various information flows, is shown in Figure 4.

The highest measurement accuracy is provided by mine-surveying; however, its use imposes a number of limitations, because, to obtain data required, the direct presence of a person in the production environment is needed, which conflicts with the requirements for the continuity and safety of mining robotized operations. Nevertheless, the mine survey supplies an array of coordinates on the basis of which a primary elevation map is formed, which can be used as a wireframe surface of an infrastructure system, which is necessary to determine the spatial position of the technological zones.

Satellite or aerial imagery does not have any significant restrictions on its use; however, it has the worst indicators of accuracy and measurement frequency. Nevertheless, the use of optical images is necessary to highlight the clear boundaries of technological zones that act as space constraints when dividing a plane into atomic elements (primitives).

The onboard sensors provide the highest data update rate with average accuracy and allow the online registration of coordinates and the state of agents of the technical agent system. This, accordingly, makes it possible to quickly determine the boundaries of technological zones as well as the accompanying geometric and mechanical characteristics of measurements of the surface of technological zones, which are, in fact, used to form a dynamic digital model of the technological environment. Table 1 presents some comparative characteristics of the listed sources of information.

In general, the process of a digital model for technological environment (infrastructure system) construction includes the following stages:

1. Aggregation of information in a distributed data warehouse.
2. Determination of coordinates of the initial boundaries of technological zones.
3. Setting of the algorithm for calculating the boundary points of the technological zone (contour restoration).
4. Accumulation of coordinates of moving mobile agents and the formation of a cloud of points in the space of technological zones.
5. Partitioning the space of technological zones into atomic elements (tessellation paving).
6. Formation of a digital model of technological zones.
7. Updating the coordinates of the boundaries of technological zones (delineation).

Stages 3–7 are repeated with a period, depending on specific technological processes.

## 4. The Structure of a Dynamic Digital Model

**Figure 5:**
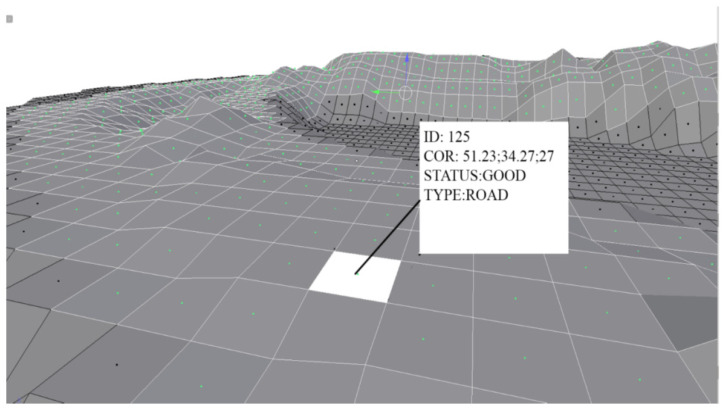
Graphical representation of an open-pit mines description using atomic elements (in this case, squares).

The key element of the dynamic digital model construction is the tessellation paving procedure—filling the selected limited technological space with atomic elements, which are geometrically regular polygons specified by the coordinates $\left\{X_{i},Y_{i},Z_{i}\right\},\;i=0,1,\ldots ,k;k$ is the number of vertices of the geometric figure. In this work, equilateral triangles, squares, and regular hexagons were used as geometric images of atomic elements and the main computational experiments were carried out on various fragments of a technological quarry roads. These geometric figures form a framework of a dynamic digital model of the quarry technological environment, which is an array of ordered and structured data, the dimension of which can change, depending on the specified constraints: given accuracy of solving problems, existing measurement error, the required speed of calculating the trajectory for each mobile agent, and the degree of reliability in the calculation of the boundary points of technological zones. Figure 5 gives a landscape illustration of the digital 3D model.

In fact, the digital model is a collection of dynamic databases: $D_{1},\;D_{2},\ldots ,D_{M}$, where M is a set of fragments of technological zones, each of which is a tensor of atomic elements $D_{i}=\left\{e_{11},e_{22},\ldots ,e_{ij},\ldots ,e_{pq}\right\}$, where p and q set the number of elements describing the roadway of rectangle form along the X and Y coordinates, respectively. Obviously, in the case when the technological fragment has an irregular shape (this is a typical situation), p and q for each column and each row of the table may be different. Each atomic element $e_{ij}$ is specified in the space of attributes—parameters $e_{ij}=\left\{a_{ij}\left(1\right),a_{ij}\left(2\right),\ldots ,a_{ij}\left(m\right)\right\}$, which define all its characteristics, such as the following:

- Unique identification number;
- Belonging to a certain technological zone;
- Type of road surface;
- A set of coordinates of the vertices and the center of an atomic element;
- $C_{ij}$—quality index of an atomic element (calculated coefficient).

The indicator $C_{ij}$ plays a key role in the process of automatic formation of routes for the movement of unmanned mobile agents.

**Figure 6:**
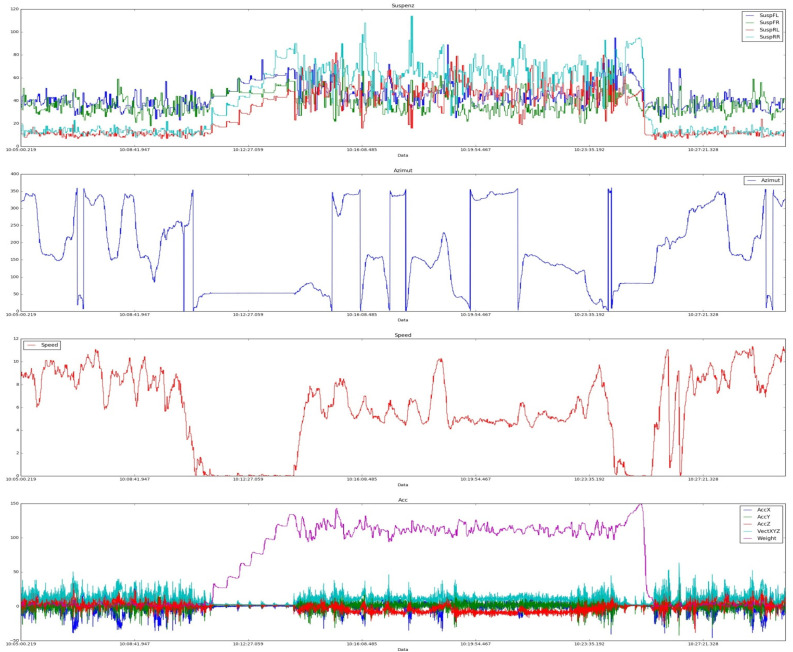
Examples of data of onboard telemetry of heavy dump trucks.

This coefficient varies in the range [0–1] and is calculated using a set of fuzzy rules, such as the following:

- $f_{1}$ is the location within the technological space (for example, “close–far” from the border of the technological zone);
- $f_{2}$ is the location of the plane of an atomic element in space (the best option is $Z_{i}=Z_{j},\;i,j=1,k\;$ for all vertices of an atomic element);
- $f_{3}\;$ is an evaluation function characterizing the current state of an atomic element (stone, pit, pothole, track trough, etc.), which is formed on the basis of expert statistical analysis of data received from onboard sensors (examples of such implementations are shown in Figure 6). Data from the load sensors on the springs of all wheels of the dump truck, as well as the speedometers, accelerometers, and inclinometers are used. As a result, for each atomic element using a fuzzy rule base, the integral estimate $C_{ij}=F\left\{f_{1},f_{2},f_{3}\right\}$ is calculated.

It is interesting to note that this may be suggested as a mechanism for solving a more general problem, namely, determining the optimal set of attributes describing the entire set of elements of heterogeneous multiagent systems—$A_{T},A_{I},A_{G}$, for example, a fragment of an excavator site, a geostructural block, and a dump truck (excavator). When solving this problem, it is only necessary to correctly combine and synchronize all the data available for registration according to the scheme described in Section 2. Further, any effective algorithm for constructing decision trees can be used in order to determine a set of attributes that will minimize the entropy introduced by the digital twin, depending on the amount of information involved in its construction.

## 5. Computational Experiments with a Digital Model—Determination of Model Parameters

**Figure 7:**
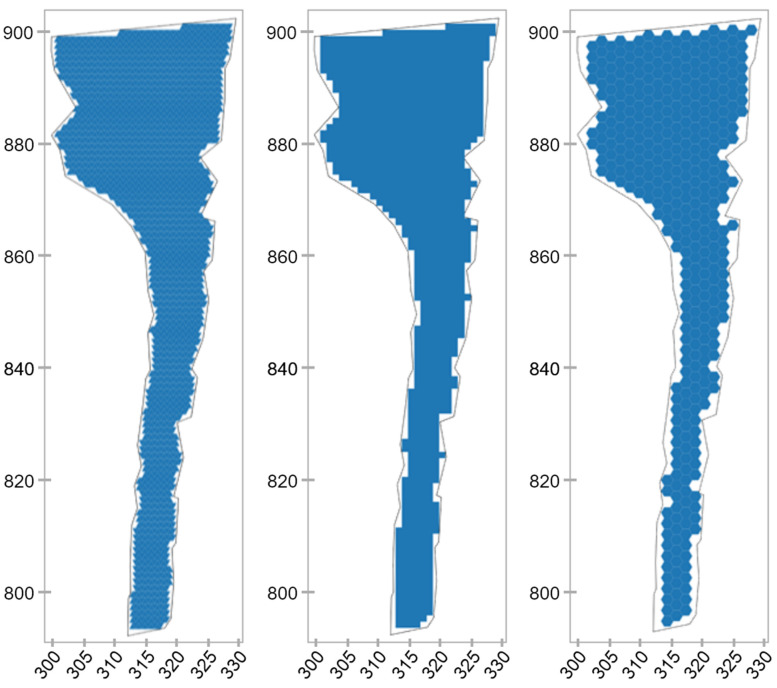
Variants of tessellation paving a fragment of a technological road with various geometric primitives with a fixed edge size (r = 1 m).

As noted earlier, the procedure for filling a technological zone with the correct polygons is an important stage in the construction of a digital model, since it defines the spatial characteristics of atomic elements. However, this task is quite trivial and is of no independent interest. Figure 7 shows several options for tessellation, paving a real fragment of the roadway using atomic elements of three types (triangle, square, hexagon). An important parameter that must be taken into account when using the model to solve practical problems is the area of this technological zone (equal to 1244 m^2^). When calculating the areas of figures of complex shape, the boundary points of which i=1,n were determined in a different way, the well-known Gauss formula was used:

(1) $$S=\frac{1}{2}\left|\overset{n-1}{\underset{i=1}{\sum }}x_{i}y_{i+1}+x_{n}y_{1}-\overset{n-1}{\underset{i=1}{\sum }}x_{i+1}y_{i}-x_{1}y_{n}\right|$$

Table 2 presents the main characteristics that illustrate this process. Obviously, for all types of figures,

(2) $$\underset{r\to 0}{\lim}\left\{S_{z}-S^{*}\right\}\to 0\;$$

However, it is also obvious that in this case, the number of atomic elements will tend to infinity, which will make the model unusable.

In the table above:

$S_{z}$—the area of the effective addressable space of the road surface;

$S^{*}$—the area of the technological road roadway obtained from the mine survey.

**Figure 8:**
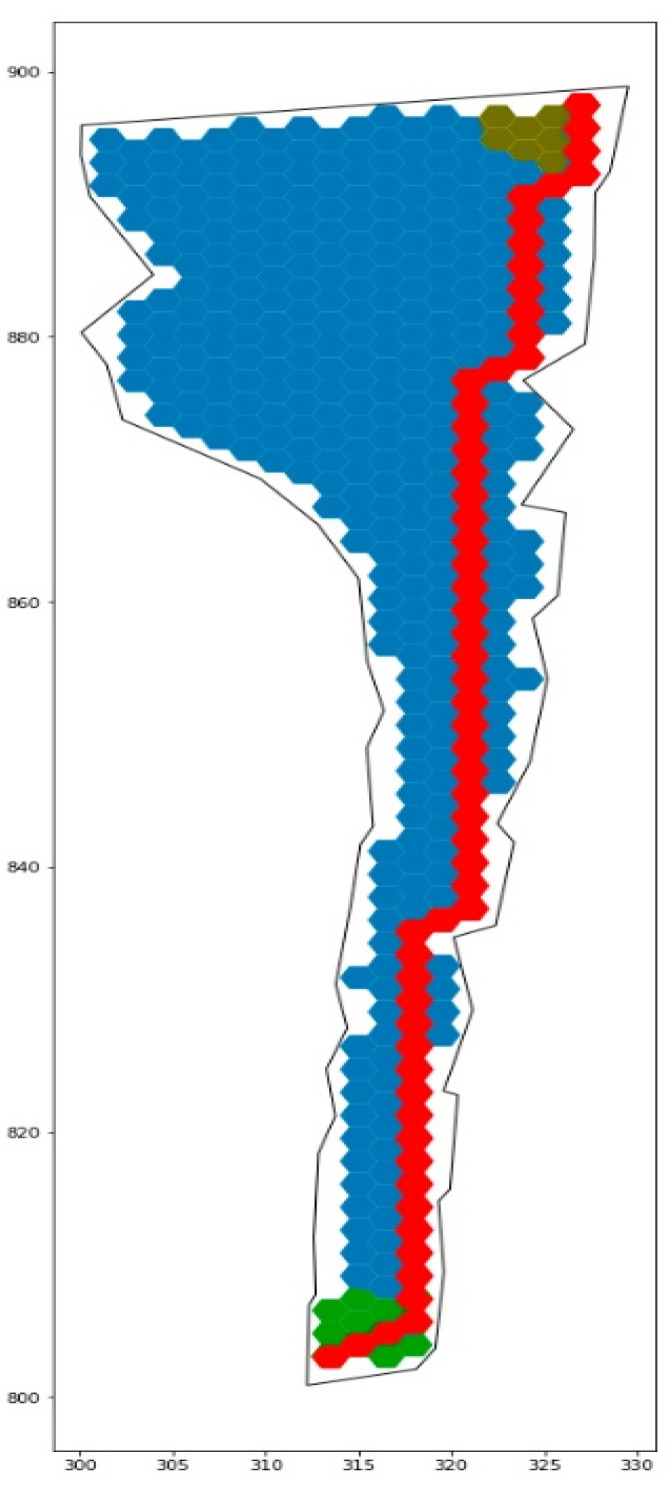
Optimal trajectory of autonomous agent moving.

The main task, for the solving of which the construction of a digital infrastructure model is carried out, is the task of choosing the optimal trajectory for the movement of an autonomous mobile device between the given points, which are determined by the route appointed. An example of solving this task is illustrated in Figure 8.

To solve the task, we used a well-known and quite effective optimization algorithm in the search space—A*, which implements the mechanism of informed greedy search, minimizing the following function:

(3) $$F=\overset{l_{f}}{\underset{k=l_{0}}{\sum }}C_{k}\left(l_{ij}\right)\to min\;$$

In addition, it should be noted that the accuracy of the operational restoration of the boundary points of the technological zone (“contouring”) is a serious problem without additional and very expensive surveying and aerial photo surveys. We have implemented a workable algorithm that allows us to build an external contour of the technological zone based on the available telemetric positioning data and the alpha–SHAPE procedure. However, the accuracy of this computational procedure significantly depends on the shape of the technological zone. As a parameter for estimating the accuracy of “contouring”, we used the area $\hat{S}$, calculated on the basis of the reconstructed boundary points (according to the Gauss formula):

(4) $$A_{L}=1-\hat{S}/S^{*}\;$$

As a result of statistical processing of large amounts of data using technological zones of various geometric shapes and sizes, a number of fairly simple but useful models were built that allow predicting the time spent on calculating trajectories using the available data, according to the accuracy of “contouring”, the degree of tessellation paving, and the size of the atomic element. General view of the constructed models:

(5) $$\gamma_{t,S,H}=\beta_{1}x_{1}+\beta_{2}x_{2}+\beta_{3}x_{3}\;$$

where $\gamma_{t,s,h}$ is forecast trajectory calculation time for one technical agent; t,s,h are types of atomic elements (triangle, square, hexagon); $x_{1}:l$ is length of the edge; $x_{2}:1-\hat{S}/S^{*}$ is a parameter that characterizes the accuracy of “contouring”; $x_{3}:S_{z}/S^{*}$ is completeness of filling the technological zone with atomic elements; $\beta_{1}$,$\;\beta_{2},\;\beta_{3}$ are coefficients.

## 6. Results

In accordance with the proposed computational models for building an infrastructure agent of a quarry, we considered a fragment of the technological environment, the characteristics (meaning the area) of which are presented in Table 3, and its visual interpretation is reflected in Figure 8.

Table 3 also shows the characteristics of the algorithm when determining the trajectory of a mobile agent between some boundary points of the route while using models with different geometric parameters. From the results of the calculations, it can be seen that the computation time (correlated with the complexity of the computed trajectory) and the accuracy of the road surface approximation are critical parameters that determine the structure of the model. It can be seen that when choosing an atomic element that is too small, the calculations can take several hours. On the other hand, a tessellation paving accuracy of less than 80% significantly reduces the space for the efficient movement of an autonomous vehicle and its safe maneuver.

For each of the space-partitioning options, in accordance with (3), the optimal trajectory of the autonomous agent (dump truck) was calculated, as shown in Figure 8, which, in general, showed the viability of solving the agent management problem using the proposed space-compaction method.

**Figure 9:**
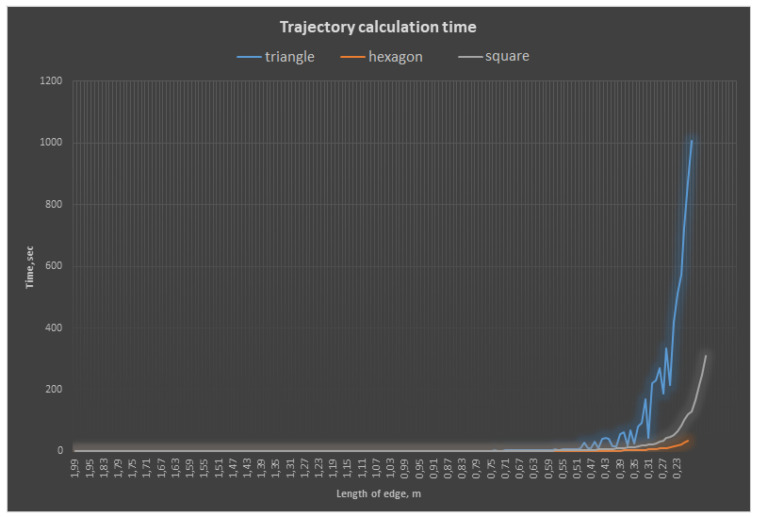
Dependence of the trajectory calculation time on the type and size of the atomic element.

Figure 9 illustrates the exponential increase in the time and complexity of calculations with a decrease in the size of the primitive edge.

As a result of computer modeling, which was carried out using fragments of roads and other technological zones of various shapes, it was possible to establish the coefficient, with the help of which we estimated the accuracy of the procedure, which lies in a fairly wide range of 0.05–0.4. It is clear that if the delineation error can reach 40% of the area, then the issues of choosing the size of the atomic element fade into the background and the maximum possible (based on the dimensions of mobile agents) size of the atomic element (2 m) can be used.

The constructed models proved to be very useful when setting up digital models and creating a Digital Twin of the technological zones of the quarry.

## 7. Discussion

It is important to focus once again on the key points of this study as a whole and briefly discuss the prospects for its further development. At the beginning, it should be emphasized that despite a number of specific features inherent in mining production (in our case, an open mining method is considered), this object is a typical example of a complex continuous production that integrates different types of technological processes that have a stochastic, nonlinear nature; moreover, these occur in a nonstationary environment. In our opinion, the construction of adequate and functional dynamic 3D models (Digital Twin) is a bridge to the creation of unmanned complex industrial facilities in metallurgical, chemical, energy, and other fields. Since there are practically no examples of the development of Digital Twins of continuous dynamic processes in the scientific literature today, it seems to us that such studies are relevant from a scientific and practical point of view.

The idea of using digital twins and switching to Industry 4.0 technologies in general implies a datacentric architecture of systems, with a sharp increase in the volume of produced and processed data, as well as a significant increase in the accuracy of solutions based on such data. At the same time, the problems of the appearance of a combinatorial explosion (the required computing power and the speed of the system as a whole) become obvious, the elimination of which are not possible solely due to the methods of transmitting, storing, and processing information. Of course, it should be recognized that a significant part of the study is devoted to the description of the developed tool, with the help of which it is possible to analyze the work of the built digital twin. The key idea of the work is to search for rational approaches to the densification of the search space of states describing a heterogeneous industrial environment.

In this regard, we have implemented the idea of building a multiagent hybrid system in which agents (belonging to three areas that are obvious and natural for a mining enterprise: technological infrastructure, technical facilities and robots, geostructure of the deposit) dynamically interact, using the same operational sensory and conditionally stationary data according to the rules formulated by us, and are original. In our opinion, they are also quite adaptable to other complex objects, where it is also easy, based on their basic technologies and common sense, to make the initial decomposition into subsystems.

This article provides only one example of the search for the optimal spatial and parametric structure of the elements of these multiagent systems. It should also be noted that the real situation, in particular, in the mining industry, is unfortunately still extremely far from the full-scale industrial operation of control systems based on digital duplication. The implementation of some of our ideas assume the presence of certain technological solutions of Industry 4.0 in enterprises, such as a reliable data transmission network with high bandwidth, the implementation of individual robotic elements, etc. We believe that our work provides a certain vector of development—describing the specific functionality of digital twins and Industry 4.0 technologies—that mining and other complex industrial enterprises that integrate different technologies should strive for.

## 8. Conclusions

We note the main issues raised in this paper and the most important results obtained by us.

1. A brief overview of the landscape defining today’s approaches to the implementation of open-pit mining technologies within the framework of the functioning of modern automated process control systems with the use of robotic or fully autonomous mining and transport equipment was performed.
2. The analysis of modern approaches to the implementation of autonomous haulage systems allows us to conclude that a wide range of predictive analytics and machine learning methods have been successfully used to solve the problems of coordinating actions and managing the coordinated movement of autonomous transport objects.
3. It was noted that industrial transport and technological systems, which certainly include mining technologies of open-pit mining, have a number of distinctive features associated with the peculiarities of the operating environment and the tasks to be solved. This refers to heterogeneous autonomous systems that must function in a constantly changing technological environment.
4. Taking into account the multicriteria and nonstationarity of transport and technological processes in quarries, it is proposed to move to a new qualitative level of modeling with the use of technological Digital Twin. The possible problems that may arise during the process of constructing this class of models in the conditions of a continuous, stochastic, and nonstationary modeling object are shown.
5. The possibilities of using modern monitoring tools, and onboard and stationary telemetry systems operating within the framework of modern automated control systems are considered.
6. An approach to modeling the processes of open-pit mining is proposed, which consists in the decomposition of the entire technological complex into three agent systems, which are defined as: technical mobile, infrastructural–technological, and geostructural.
7. A methodology and an appropriate set of algorithms have been developed aimed at integrating heterogeneous geospatial and telemetric information in order to build a digital 3D model of the technological environment of the quarry (infrastructure and technological system). The basis of the methodology is the structure of the digital model of transport and technological zones of the quarry.
8. A set of computational experiments was carried out, which confirmed the efficiency of the proposed method. Based on the analysis of the simulation results, specific recommendations can be formulated for the implementation of the proposed methodology at any mining facility with robotic equipment equipped with modern sensor equipment.

## Figures and Tables

**Table 1:** Characteristics of the sources of information.

Characteristics	Mine-Surveying	Satellite Imagery	On Board Telemetry
Accuracy	1 cm	100 cm	50 cm
Completeness of the data	High	High	Medium
Update frequency	Once a week	Discretely nondeterministic	Continuous
$\mathrm{Height}\;(Z_{i}$) accuracy	1 m	10 m	2 m

**Table 2:** Characteristics of tessellation paving with a fixed edge length.

Type of Atomic Element	Length of Edge, m	Quantity of Elements, p×q	$S_{z}/S^{*},\;\%$
square	1	1114	92.83
triangle	1	2270	94.58
hexagon	1	394	85

**Table 3:** Characteristics of the algorithm’s operation with various models.

Type of Atomic Element	Length of Edge, m	Quantity of Elements, p×q	Complexity, n	Time of Calculation, sec	Square, m^2^	$S_{z}/S^{*},\;\%$
square	2	245	54	0.041955	980	81.67
square	1	1114	111	0.375658	1114	92.83
square	0.5	4723	226	5.399732	1180.75	98.40
square	0.25	19.466	458	118.5997	1216.63	99.39
triangle	2	535	96	0.081783	1070	89.17
triangle	1	2270	192	0.660409	1135	94.58
triangle	0.5	9352	381	40.54165	1169	97.42
triangle	0.25	37.988	758	1208.144	1187.13	98.93
hexagon	2	77	28	0.019948	800.21	66.68
hexagon	1	394	60	0.088249	1023.64	85.30
hexagon	0.5	1754	121	0.874117	1139.26	94.93
hexagon	0.25	7355	247	13.87281	1194.30	99.53

## Data Availability

Not applicable.
